# Repair of ascending aorta pseudoaneurysm without circulatory arrest in redo patient

**DOI:** 10.1186/1749-7922-1-2

**Published:** 2006-03-24

**Authors:** Stefano Auriemma, Paolo Magagna, Ayman Sallam, Nicola Lamascese, Alessandro Fabbri

**Affiliations:** 1 Division of Cardiac Surgery San Bortolo Vicenza Hospital, Vicenza, Italy

## Abstract

A pseudoaneurysm of the ascending aorta is an unusual and potentially fatal complication after aortic surgical operations. TEE and CT scan are the investigations of choice. Surgical treatment is mandatory. We describe the successful management of a pseudoaneurysm of the ascending aorta, with aorto-sternal-cutaneous fistula requiring right axillary and femoral artery cannulation with Remote Access Perfusion^® ^aortic cannula (Estech^®^, California, USA). Behaving like this we avoid hypotermic circulatory arrest, provide safe reentry and prevent an impending rupture.

## Background

Mediastinal pseudoaneurysms are rare but lifethreatening complications of thoracic aortic operations [1,2]. Predisponing factors are the dissection of the native aorta, infection, connettive tissue disorders, preoperative chronic hypertension, aortic calcification and blowout of the aortotomy site [1].

A pseudoaneurysm of the ascending aorta could present itself as a pulsatile mass, angina due to graft compression, chest pain caused by local erosion, dysphagia or stridor [2].

Redo operations for large pseudoaneurysm of the ascending aorta are a surgical challenge [3]. Sternal reentry by itself can precipitate fatal haemorrhage or cerebral air embolism.

Our technique points out the use of right axillary artery cannulation and endoclamping perfusing aortic cannula to avoid hypothermic circulatory arrest [5].

This allows us to prevent an impending rupture, to repair the defect and to preserve the brain and the other organs during the procedure.

## Case report

**Figure 1:**
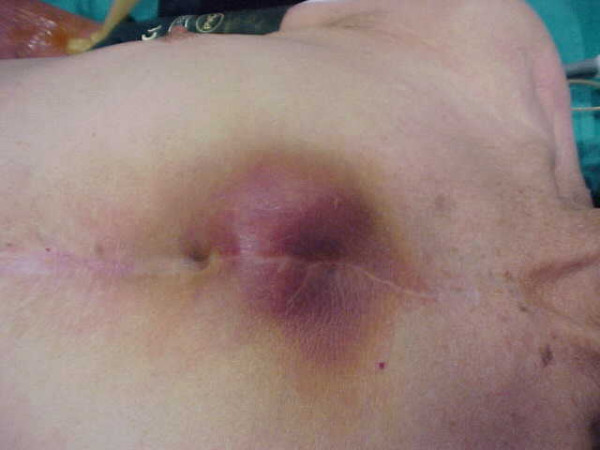
Patient's antero-lateral view showing pulsatile erythematous swelling over the upper third of the sternum.

Our case treats of a 79-years-old man who has electively undergone aortic valve and ascending aorta replacement for degenerative aortic disease using a number 25 Edwards Lifesciences porcine stentless bioprosthesis (Edwards, California, USA). He was readmitted 4 months later with fever, intermittently painful and pulsatile swelling over the upper third of the sternum that appeared over a period of 15 days and had gradually increased in size to attain its present size of 6 × 4 cm (Fig. 1).

A preoperative TEE showed a pseudoaneurysm with high flow velocity inside, developed between the prosthesis and the posterior surface of the sternum.

**Figure 2:**
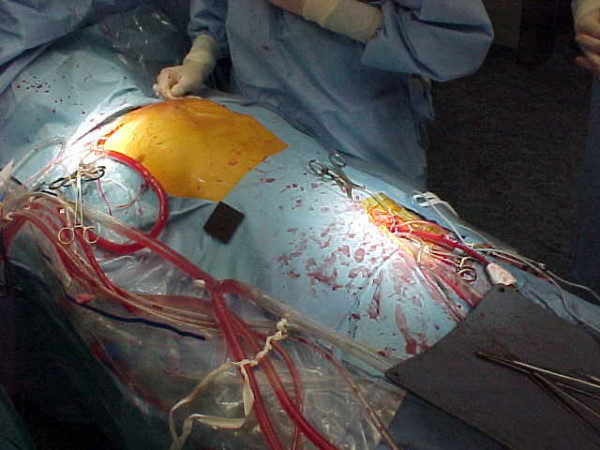
Arterial perfusion sites.

**Figure 3:**
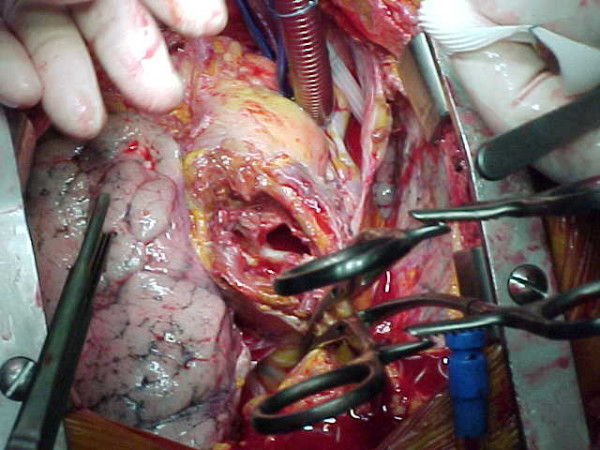
Intra-operative view showing the defect in the ascending aorta.

Moderate hypothermic bypass was instituted with cannulation of right axillary artery for antegrade cerebral perfusion and femoral artery with Estech^® ^cannula for aorta endoclamping and perfusion of lower body districts [5] (Fig. 2). The chest was opened through the previous scar reducing the CPB flows and obtaining digital control over the defect on the aorta. Subsequently a vent cannula has been placed in the right superior pulmonary vein to decompress the left side of the heart. The extrathoracic component of the pseudoaneurysm was opened and the clots evacuated. Adhesions over the right ventricle were taken down, and both pleura were opened. Clots in front of the aorta were evacuated to reveal one main defect at the previous aortotomy site measuring 2 × 2 cm (Fig. 3). The bioprosthesis was explored through the defect and was found to be normal. The defect was closed using 3 × 3 cm polytetrafluoroethylene patch.

After deairing the patient was rewarmed and the heart picked up after single electric shock in sinus rhythm. The weaning from the bypass was easy.

The patient remained hemodynamically stable on minimal inotropic support and was extubated in second postoperative day without neurologic complications.

The cause of the pseudoaneurysm appeared to be an infection at the previous aortotomy site; the cultures taken from adjacent tissues and intraluminal blood clots were positive for Staphylococcus aureus.

## Discussion

Pseudoaneurysm of the ascending aorta after aortic procedure usually occur at anastomotic suture lines or at aortotomy, aortic cannulation or aortic needle puncture sites. Most episodes have infective causes [1]

Contrast CT scanning, RMI and echocardiography are useful in the diagnosis of pseudoaneurysm of the ascending aorta. Surgical repair is mandatory [1,4].

The most important issue is to protect the brain and systemic perfusion before opening the chest, because an inadvertent pseudoaneurysm rupture during a repeat sternotomy or mediastinal dissection can lead to a catastrophic intraoperative haemorrhage.

The use of CPB with cannulation of right axillary and femoral arteries and the use of Remote Access Perfusion^® ^aortic cannula have helped us to prevent an impending rupture. This has avoided deep hypotermic circulatory arrest and has allowed us to effect an adequate and safe repair without time limits [5].
